# Biochar addition can negatively affect plant community performance when altering soil properties in saline-alkali wetlands

**DOI:** 10.3389/fpls.2024.1347658

**Published:** 2024-05-16

**Authors:** Ziyi Wang, Mengxuan He, Xueqiang Lu, Zirui Meng, Jie Liu, Xunqiang Mo

**Affiliations:** ^1^ School of Geographic and Environmental Science, Tianjin Normal University, Tianjin, China; ^2^ College of Environment Science and Engineering, Nankai University, Tianjin, China; ^3^ State Key Laboratory of Herbage Improvement and Grassland Agro-ecosystems, Center for Grassland Microbiome, College of Pastoral Agriculture Science and Technology, Lanzhou University, Lanzhou, China

**Keywords:** biochar, coastal saline-alkali soils, soil properties, plant community performance, community stability

## Abstract

Biochar is a widely proposed solution for improving degraded soil in coastal wetland ecosystems. However, the impacts of biochar addition on the soil and plant communities in the wetland remains largely unknown. In this study, we conducted a greenhouse experiment using soil seed bank from a coastal saline-alkaline wetland. Three types of biochar, including *Juglans regia* biochar (JBC), *Spartina alterniflora* biochar (SBC) and *Flaveria bidentis* biochar (FBC), were added to the saline-alkaline soil at ratios of 1%, 3% and 5% (w/w). Our findings revealed that biochar addition significantly increased soil pH, and increased available potassium (AK) by 3.74% - 170.91%, while reduced soil salinity (expect for 3% SBC and 5%SBC) by 28.08% - 46.93%. Among the different biochar types, the application of 5% FBC was found to be the most effective in increasing nutrients and reducing salinity. Furthermore, biochar addition generally resulted in a decrease of 7.27% - 90.94% in species abundance, 17.26% - 61.21% in community height, 12.28% - 56.42% in stem diameter, 55.34% - 90.11% in total biomass and 29.22% - 78.55% in root tissue density (RTD). In particular, such negative effects was the worst in the SBC samples. However, 3% and 5% SBC increased specific root length (SRL) by 177.89% and 265.65%, and specific root surface area (SRSA) by 477.02% and 286.57%, respectively. The findings suggested that the plant community performance was primarily affected by soil pH, salinity and nutrients levels. Furthermore, biochar addition also influenced species diversity and functional diversity, ultimately affecting ecosystem stability. Therefore, it is important to consider the negative findings indirectly indicate the ecological risks associated with biochar addition in coastal salt-alkaline soils. Furthermore, *Spartina alterniflora* was needed to desalt before carbonization to prevent soil salinization when using *S. alterniflora* biochar, as it is a halophyte.

## Highlights

- Biochar addition increased soil pH, available potassium and decrease soil salinity (expect for 3% SBC and 5%SBC).
- Biochar addition changed plant morphological traits attributed to alterations of soil properties.
- Biochar addition altered species and functional diversity, thereby changing stability.
- Biochar addition in salt-alkaline soils carries the potential for ecological risks.

## 1 Introduction

Soil salinization is a widespread issue that poses serious threats to the stability of wetland ecosystems (He et al., 2014). The expansion of saline soil has attracted increasing great attention, and many countries have undertaken ecological restoration projects to restore the deteriorated soils (Nouri et al., 2017; Chávez-García and Siebe, 2019; Xu et al., 2020). Recently, biochar exhibits considerable potential as an effective tool for remediating degraded soils (El-Naggar et al., 2019; Yang et al., 2019; Yuan et al., 2023);. Biochar, a porous solid residue rich in carbon obtained through high temperature pyrolysis (<700°C) in oxygen-limited conditions (Saifullah et al., 2018; Tang et al., 2020). In reality, the addition of biochar to the soil could improve soil quality due to its unique characteristics, including porous structure, rich surface charges and functional groups (Thomas et al., 2013; Hammer et al., 2015; Yuan et al., 2019). Studies have already proved that biochar benefits when acting as a soil remediation, such as reducing soil heavy metals, promoting cation exchange capacity (CEC) and soil nutrients (Gundale et al., 2016; Luo et al., 2016; Wang et al., 2022). More importantly, it can also influence plants which are highly sensitive to soil properties.

Previous evidence has already demonstrated that using biochar has the ability to indirectly affect plants performance, mainly by altering the soil physicochemical properties (Akhtar et al., 2015; Roberts et al., 2015; Farooq et al., 2020). However, the impact of biochar addition on plant growth and performance largely depends on the characteristics and physicochemical properties of the biochar, as well as its interactions with the soil (Haider et al., 2022; Major et al., 2010). For instance, Ochiai et al. (2021) found that applying manure biochar increased the biomass of oat plants more than wood biochar, possibly due to the favorable properties of manure biochar such as its labile-C and -N content and high pH. Biochar derived from plant residues is commonly used as a soil conditioner rather than a fertilizer due to its low leachable nutrient content (Haider et al., 2022; Yuan et al., 2019). Furthermore, the effects of wood biochar and straw biochar on maize growth were positive in slightly acidic soils but had no effect in alkaline soil (Bornø et al., 2018). The high pH of biochar is widely recognized for its effectiveness in improving acidic soils, its efficacy in alkaline soils is still a matter of debate. Accordingly, it is important to consider the significant interaction between soil type and biochar.

Researches have shown biochar application to be benefit plant growth, biomass and plant nutrient uptake in saline- alkali soil, as it can efficiently boost soil nutrient and reduce soil salinity (Cui et al., 2021; Li et al., 2021; Zhang et al., 2022). But biochar addition can potentially have negative effects on soil and the growth of plants. For instance, the combination of biochar and P fertilization can lead to P precipitation or sorption reaction in saline sodic soil, which reduced plant P availability (Xu et al., 2014). In addition, when lignocellulosic biochar is mixed with soil at a rate of 10%, it has been observed to significantly impede the height of plants and the weights of *Miscanthus* (He et al., 2020). These conflicting findings point to the effectiveness of biochar addition in saline-alkali soil remains uncertain. Furthermore, changes in soil structure and quality caused by biochar addition can impact the composition and succession of plant communities (Gundale et al., 2016). Plant community ecological indicators, such as species richness, diversity index and evenness index, provide a more comprehensive evaluation of soil ecosystem health than individual plant growth indicators (Meng et al., 2023). However, the impacts of biochar on plant communities are still unclear, which constrains our ability to restore vegetation in coastal saline-alkali wetlands.

Tianjin Binhai Coastal Wetland, a typical representative of coastal saline-alkaline wetland in China (average salt content of 1% - 4%), is dominated by halophytes, such as *Suaeda salsa, Phragmites australis* and *Suaeda glauca* (Mo et al., 2010). Considering the low vegetation coverage and homogenous vegetation structure in this region, to achieve vegetation restoration by soil remediation has become the focus of attention. Biochar as a conditioner has been used for soil remediation in coastal saline-alkaline wetlands (Zhang et al., 2022). However, the effects of biochar on performance of plant communities in coastal saline-alkali wetlands remain uncertain.

Therefore, to assess the impacts of biochar management on soil physicochemical properties and plant community performance, a greenhouse experiment was conducted. The experiment included a control group (CK) without biochar addition, as well as three different levels of biochar produced from *Juglans regia* (JBC), *Spartina alterniflora* (SBC) and *Flaveria bidentis* (FBC) (1%, 3% and 5%; weight ratio) added to the soil. The choice of *Juglans regia* for biochar production was based on its common availability. While the invasive plants, *Spartina alterniflora* and *Flaveria bidentis* were selected for their high biomass productivity, making them suitable for biochar production in coastal China. These invasive plants also provided a source of the invasive species in the wetland and could potentially help control invasions. The specific objectives were to reveal: 1) the alterations in soil physicochemical properties with biochar addition; 2) the effects of biochar on the morphological traits of the plant community; 3) the effects of biochar on community diversity and stability. The research study aims to investigate the feasibility of utilizing biochar obtained from invasive species for improving the ecological restoration of a wetland ecosystem.

## 2 Materials and methods

### 2.1 Soil seed bank collection and biochar preparation

The soil seed bank utilized in this research was collected from a saline-alkali wetland in Tianjin (39°13′N, 117°2′E), located in a semi-humid and semi-arid continental monsoon climate zone. The average temperature in this area is 12.3°C and the average precipitation is 566.0 mm. The soil in this area is affected by salinization, resulting in a high salt content (1% to 4%). This salinization is caused by the infiltration of seawater and underground brine. Moreover, with high soi pH indicating that it is a typical saline-alkali soil. The pH was 8.29 ± 0.11, electrical conductivity (EC) was 2273.41 ± 452.38 μs/cm. For sampling, ten sampling plots (10 m × 10 m) were taken every 600 m long along the coastline, covering an area of 100 m in width and seven sampling quadrats (1 m × 1 m) were selected from each plot. The soil samples were taken randomly from each quadrat, up to a depth of 10 cm and thoroughly mixed after removing debris and litters (Meng et al., 2023).

In this experiment, *Juglans regia*, *Spartina alterniflora* and *Flaveria bidentis* were applied as feedstocks to produce biochar. According to previous studies, biochar produced at high temperatures (≥550°C) has lower levels of toxic functional groups, such as carboxylic acids, amines and phenols (Tang et al., 2020). Therefore, our study involved air-drying the plant materials followed by charring them at a temperature of 550°C within a portable charring furnace possessing multifunctional capabilities under conditions devoid of oxygen. The produced biochar was initially passed through a 2 mm sieve and then used for soil analysis. The physicochemical properties of three kinds of biochar demonstrated distinct variations (**Supplementary Table S1** and **Supplementary Figure S1**).

### 2.2 Experimental design

The research utilized a plastic tray measuring 37 cm × 30 cm × 7.5 cm for the purpose of fitting and restraining 6 kg of pre-prepared soil. The previous studies showed that biochar addition at an optimal level of 5% or less could effectively improve degraded coastal soil (Zheng et al., 2018). Thus, the coastal soil samples were homogeneously mixed with the *Juglans regia* biochar (JBC), *Spartina alterniflora* biochar (SBC) and *Flaveria bidentis* biochar (FBC) respectively at rates of 1%, 3% and 5% (weight ratio; w/w). These mixed samples were labeled as 1% JBC, 3% JBC, 5% JBC; 1% SBC, 3% SBC, 5% SBC; 1% FBC, 3% FBC and 5% FBC, which brought a total of ten treatments with seven replicates for each. Additionally, the soil without biochar addition was used as a control (CK). The plant community analyzed in this study were that spontaneously germinated in soil seed bank. To ensure optimal conditions, water was added every two days to maintain 70% WHC. After a period of 90 days, the plant communities were harvested. The experiment with a daytime temperature ranging from 22°C to 25°C, while the nighttime temperature ranged from 8°C to 10°C, the light intensity was 50 klx, and the light duration was 12h (Zeb et al., 2022).

### 2.3 Soil properties analysis

The pH of the soil was measured using a pH meter (PHSJ-3F, INESA) with a soil-to-water ratio of 1:2.5. Soil salinity was determined by the mass method (LY/T1251–1999) (NFGA, 1999). The measurement of soil organic matter (SOM) was carried out using the sulfuric acid-potassium dichromate oxidation method (NY/T1121.6–2006) (MOA, 2006). The analysis of total nitrogen (TN) was analyzed through the Kjeldahl digestion procedure (Bremner, 1965). Total phosphorus (TP) was determined via the perchloric acid-sulfuric acid digestion method (Olsen et al., 1954). The soil ammonia nitrogen (NH_4_^+^-N) contents were determined with 1 mol L^-1^ KCI extracts and analyzed AA3 automated flow injection analysis (Auto Analyzer 3, Seal). Available phosphorus (AP) was extracted with 0.5 mol L^-1^ NaHCO_3_ and measured using the molybdenum-antimony resistance colorimetric method (HJ 704–2014) (MEPRC, 2014). Soil available potassium (AK) was extracted with 1 mol L^-1^ NH4OAc at a solution-to-soil ratio of 10:1 and measured by ICP-OES.

### 2.4 Analysis of plant community traits

At the time of harvest, we used rulers and vernier calipers to measure the stem diameter and height of each individual plant species. We also carefully identified the plant species and recorded the number of plant individuals found in each plot, then cut the plant shoots with scissors. The roots were carefully removed from the soil and then cleansed with distilled water. Following this, we employed a root analyzer to examine fine roots that were less than 2 mm in diameter. Then we were able to obtain morphological characteristics of the root, including length, surface area, volume, and average diameter. Subsequently, we placed the roots in a drying oven at 72°C until they achieved a constant weight, using a balance to measure the dry weights. Furthermore, calculating the specific root length (SRL, cm mg^− 1^), specific root surface area (SRSA, cm^2^ mg^− 1^) and root tissue density (RTD, mg cm^− 3^) based on dry mass (Hajek et al., 2013).

### 2.5 Statistical analysis

The study analyzed the variations in soil physicochemical properties and plant community traits using one-way ANOVA, followed by the LSD test. The species diversity was measured using the Shannon-Weiner diversity index, Pielou’s evenness index and Simpson diversity index, based on the abundance of species. The community functional diversity was assessed by calculating indices including the Function divergence index, Function dispersion index and Rao’s quadratic entropy. These indices were based on plant height, plant stem diameter, shoot biomass, root biomass, and water content of plant tissue. In addition, differences in species composition were evaluated through the non-metric multidimensional scaling (NMDS) technique, which aids in reducing the data dimensionality to provide insights into the relationships between samples. The community stability was determined by using inverse of coefficient of variation ICV following the Equation 1 (Yang et al., 2011; Wang et al., 2013):


(1) ICV = μ/σ

where μ is the average relative abundance of all plant species in one particular quadrat and σ is the standard deviation for the average relative abundance of all plant species in one particular quadrat. Plant communities showing higher ICV values demonstrate superior stability in comparison to those exhibiting lower ICV values.

we employed redundancy analysis (RDA) as a statistical tool to reveal the connection between soil physicochemical properties and species composition. Additionally, we utilized structural equation models (SEM) to analysis the relationships among soil physicochemical properties, morphological traits, diversity (species and functional diversity) and community stability under the three biochar addition conditions. Functional diversity and NMDS were implemented using the vegan, FD, and ggplot2 software packages for R (version 4.2.2). The SEM were constructed using Amos 26.0 software, while other analyses were performed using SPSS v.27.0 software. The graphs were drawn by Origin 2021.

## 3 Results

### 3.1 Soil physicochemical properties

**Figure 1:**
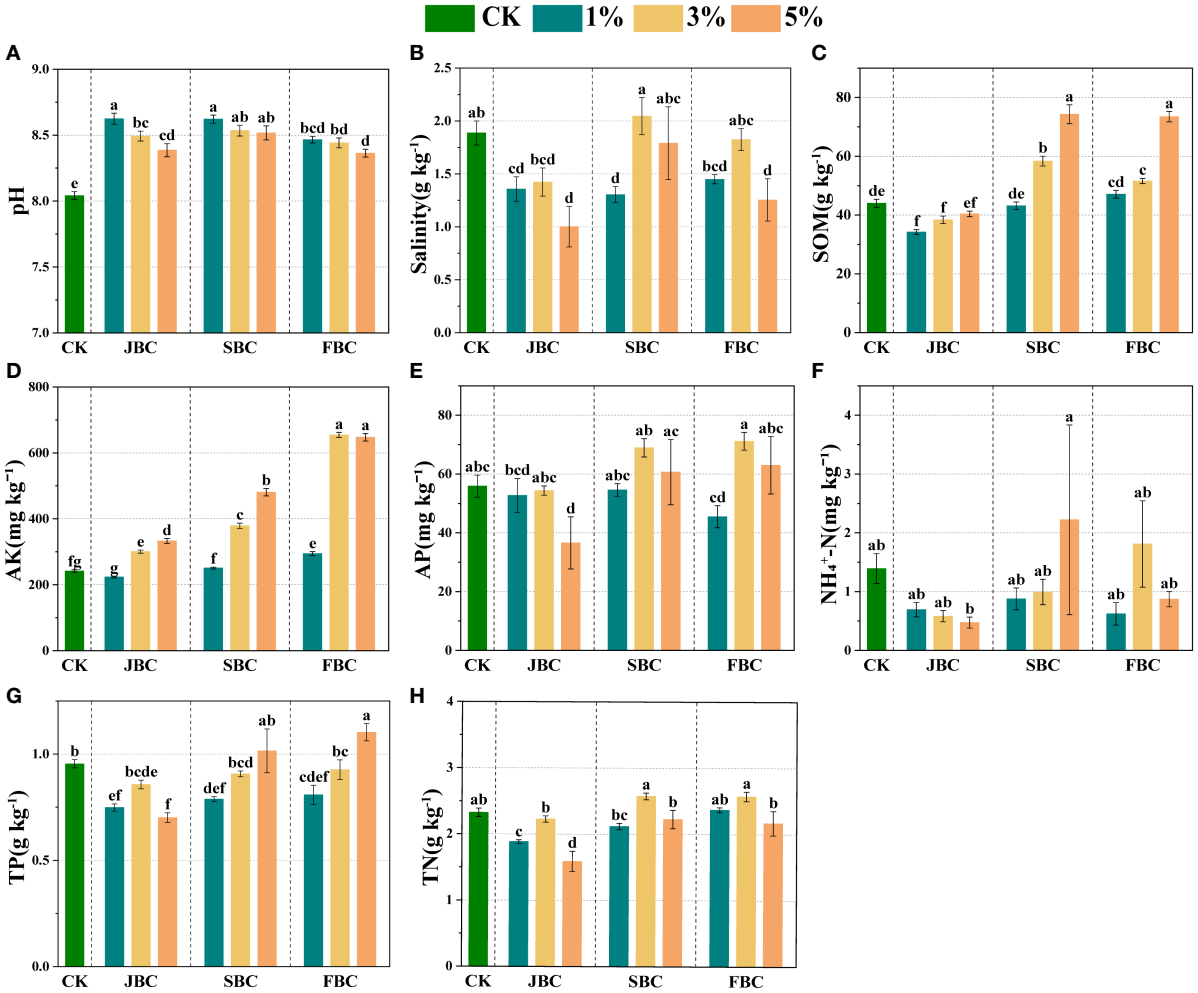
Effects of biochar on soil physicochemical properties. Bars and error bars show mean and SE (n = 7). Different lowercase letters indicate significant difference among treatments (p < 0.05). pH **(A)**, Salinity **(B)**: soil salinity, SOM **(C)**: soil organic matter, AK **(D)**: available potassium, AP **(E)**: available phosphorus, NH_4_^+^-N **(F)**: ammonia nitrogen, TP, **(G)**: total phosphorus and TN, **(H)**: total nitrogen.

In comparison to the CK treatment, biochar addition resulted in an increase in soil pH and AK by 4.00% - 7.25% and 3.74% - 170.91%, respectively (**Figures 1A, D**). Soil SOM showed a significant increase of 32.57%, 68.78%, 17.19% and 66.88% at middle and higher ratios of SBC and FBC biochar addition (**Figure 1C**). Furthermore, there was a notable increasing trend in soil AK and SOM with increasing rates of JBC, SBC and FBC addition (**Figures 1C, D**). However, biochar addition had a significant effect in reducing soil salinity by 28.08%, 46.93%, 30.89% and 33.54% at 1% JBC, 5% JBC, 1% SBC and 5% FBC, respectively (**Figure 1B**). Additionally, soil AP and TP did not show any increase with biochar additions, except for a significant increase in soil TP by 15.57% at 5% FBC (**Figures 1E, G**). Moreover, TN was decreased of 18.87% and 31.71% at 1% JBC and 5% JBC, but no significant changes were observed in NH_4_^+^-N when compared to the CK treatment (**Figures 1F, H**).

### 3.2 Morphology traits of plant communities

**Figure 2:**
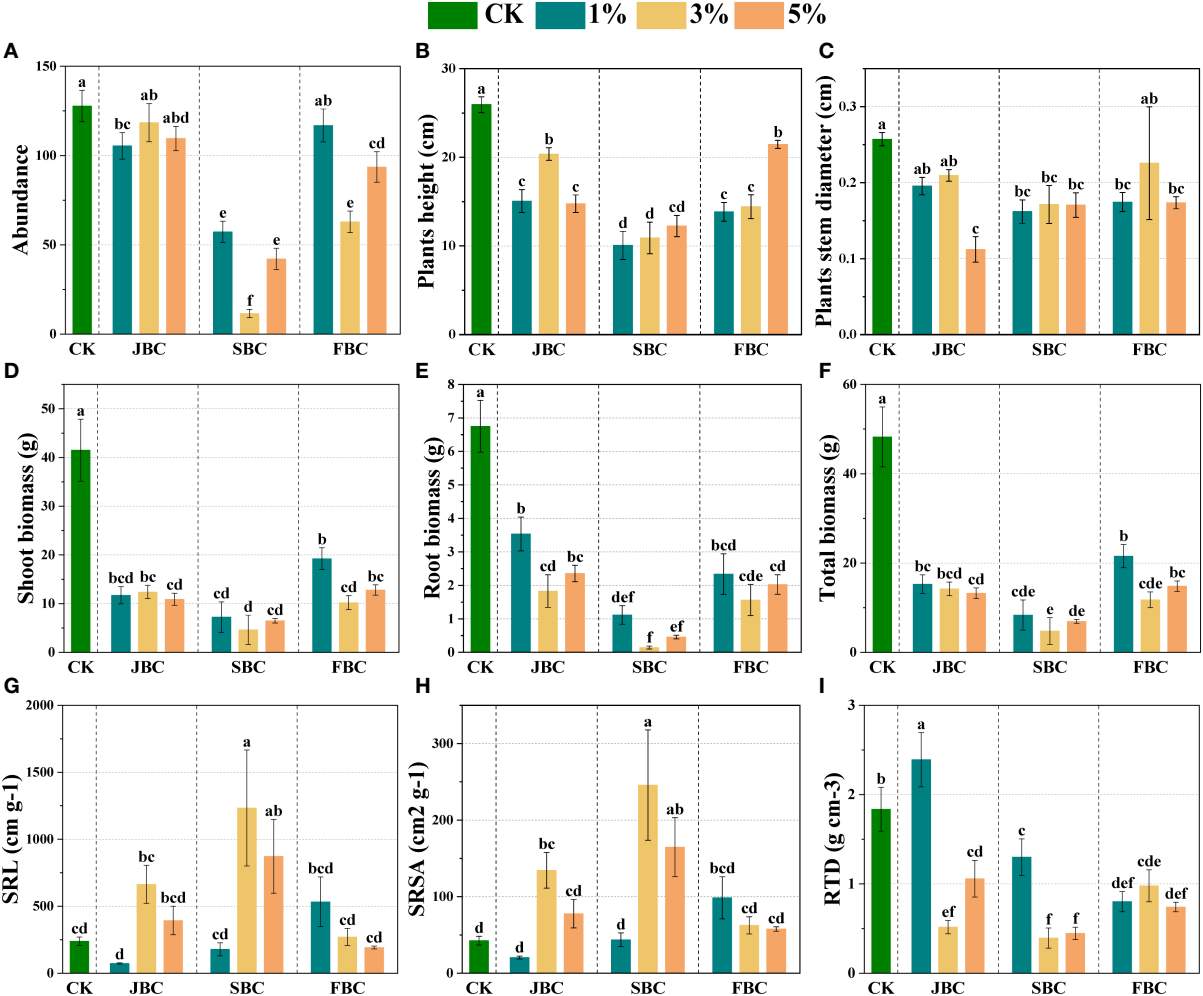
Effects of biochar on morphological traits of plant communities. Bars and error bars show mean and SE (n = 7). Different lowercase letters indicate significant difference among treatments (*p* < 0.05). Species abundance of communities **(A)**, Stem diameter **(B)**, Height **(C)**, Community biomass **(D–F)**, SRL **(G)**: specific root length, SRSA **(H)**: specific root surface area and RTD **(I)**: root tissue density.

The community morphological traits from the CK treatment differ markedly from those treated with biochar (**Figure 2**). In comparison to the CK treatment, biochar addition resulted in a reduction in species abundance by 7.27% - 90.94%, height by 17.26% - 61.21%, stem diameter by 12.28% - 56.42%, and total biomass by 55.34% - 90.11%, respectively (**Figures 2A–C, F**). Notably, such negative effect was the worst in the SBC samples. However, 3% and 5% SBC showed an increase in specific root length (SRL) by 177.89% and 265.65%, and specific root surface area (SRSA) by 477.02% and 286.57%, respectively (**Figures 2G, H**). On the other hand, the RTD increased by 30.27% at 1% JBC, while it decreased by 29.22% - 78.55% in other biochar treatments. In summary, although the biochar application positively affected SRL and SRSA in the 3% SBC and 5% SBC treatments, it may not be beneficial for overall plant growth.

### 3.3 Composition and diversity of plant communities

#### 3.3.1 Plant community composition

**Figure 3:**
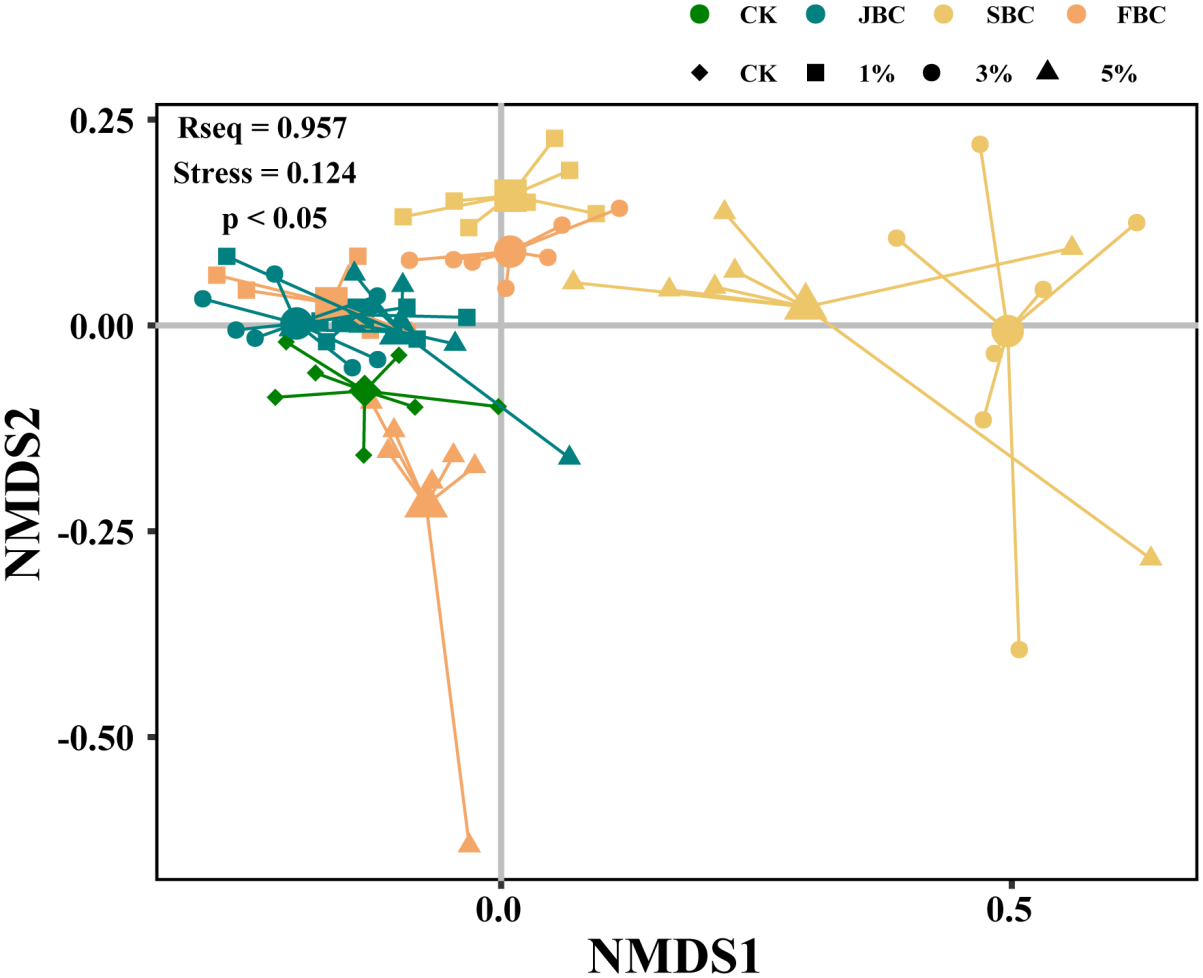
non-metric multidimensional scaling (NMDS) analysis.

The NMDS analysis revealed that the species composition varied among different ratios of the same type of biochar (**Figure 3**). However, there was no significant difference observed between JBC (1%, 3% and 5%) and 1% FBC treatments (**Figure 3**; **Supplementary Table S3**). Additionally, the relative abundance of *Setaria viridis* significantly increased under the 1% SBC and 3% FBC treatments when compared to the control treatment. Conversely, the relative abundance of *Setaria viridis* decreased, while the abundance of *Suaeda glauca* increased from 7% to 35% when treated with 5% FBC (**Supplementary Figure S4**). These findings indicate that the addition of biochar can alter the relative abundance of the plant community.

#### 3.3.2 Species and function diversity

**Figure 4:**
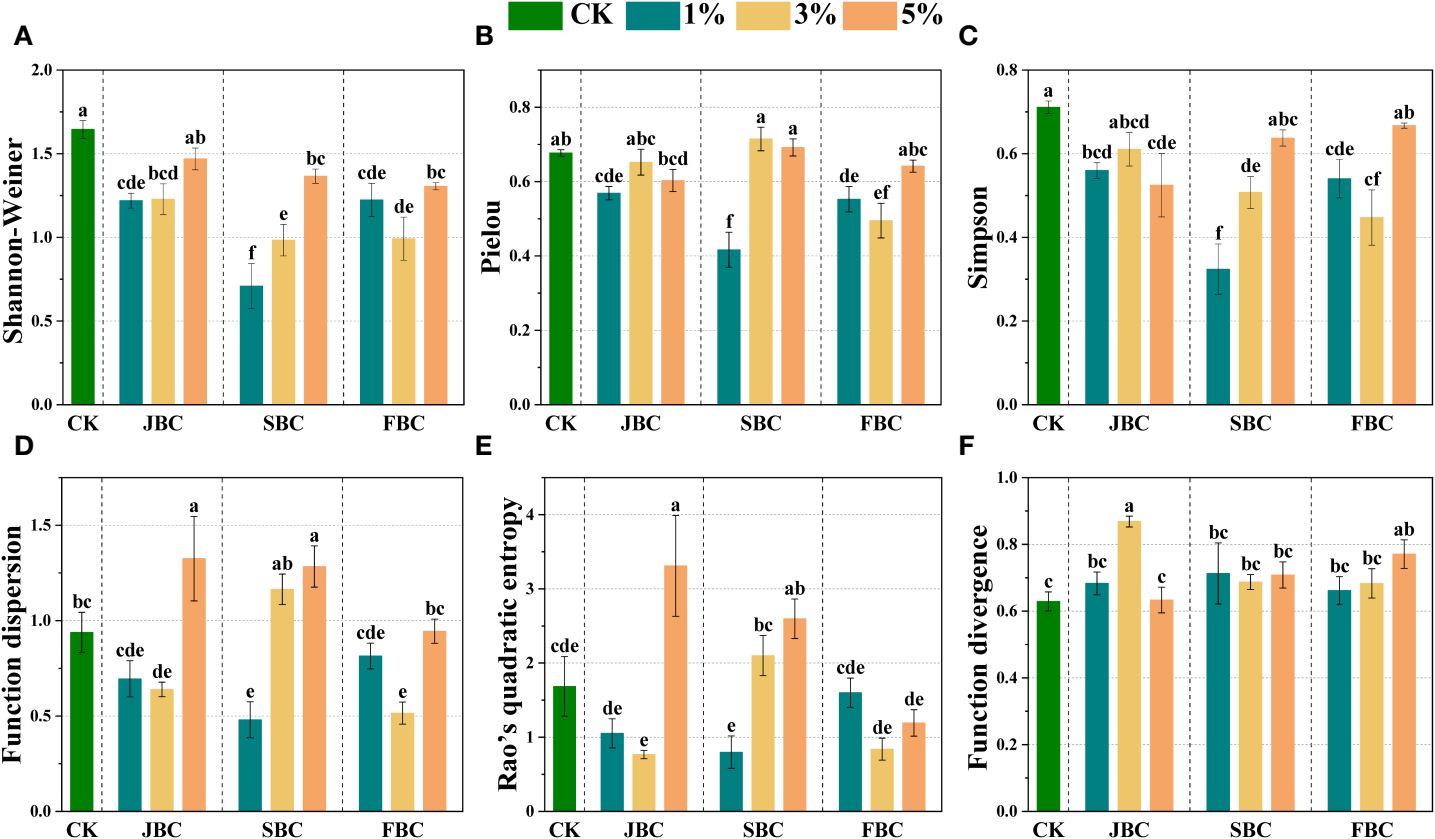
Effects of biochar on species diversity. Bars and error bars show mean and SE (n = 7). Different lowercase letters indicate significant difference among treatments (p < 0.05). Shannon-Weiner diversity index **(A)**, Pielou's evenness index **(B)**, Simpson diversity index **(C)**, Function dispersion index (FDis) **(D)**, Rao’s quadratic entropy index (RaoQ) **(E)** and Function divergence index (FDiv) **(F)**.

Most biochar addition treatments exhibited significant reductions in Shannon-Weiner, Simpson and Pielou values when compared with the CK treatment, which were lowest in the 1% SBC treatment. However, the Shannon-Wiener index and Simpson index increased with increasing SBC ratios (**Figures 4A, B**). The functional diversity of plant communities was estimated by Function dispersion (FDis), Function divergence (FDiv) and Rao’s quadratic entropy (RaoQ) (**Figures 4D, E**). The FDiv index showed a significant increase of 38.42% and 22.76% in the 3% JBC and 5% FBC treatments, respectively. The FDis index exhibited a significant increase of 62.07% and 55.46% in the 5% JBC and 5% SBC treatments, but a decline of 73.49% and 67.90% in the 1% SBC and 3% FBC treatments, respectively. Furthermore, the RaoQ index showed a significant increase of 260.75% and 146.36% in the 5% JBC and 5% SBC treatments, but a reduction of 147.43% and 142.25% in the 3% JBC and 1% SBC treatments, respectively.

### 3.4 Stability of plant communities

**Figure 5:**
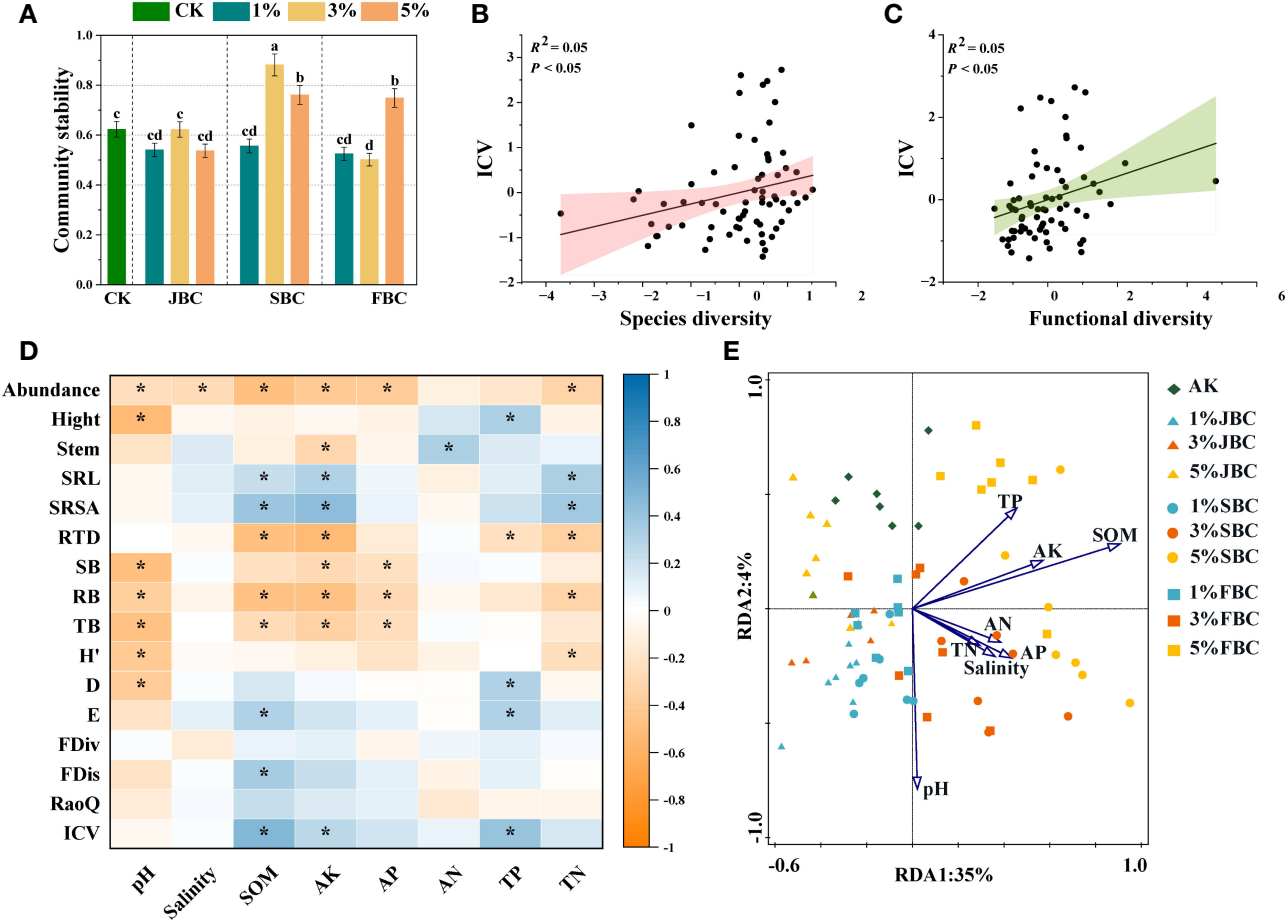
Effects of biochar on community stability **(A)**, the correlation between ICV and community species diversity **(B)** and the correlation between ICV and community functional diversity **(C)**. Spearman correlation heatmaps revealing the relationships of soil physicochemical properties, morphological traits, community diversity and stability **(D)**. RDA of soil physicochemical properties and plant community composition **(E)**. SOM, soil organic carbon; Salinity, soil salinity; AK, available potassium; AP, available phosphorus; AN, ammonia nitrogen; TN, total nitrogen; TP, total phosphorus. SRL, specific root length; SRSA, specific root surface area; RTD, root tissue density; H’, Shannon-Wiener diversity index; D, Simpson diversity index; E, Pielou’s evenness index; FDiv, Function divergence index; FDis, Function dispersion index; RaoQ, Rao’s quadratic entropy index; ICV, community stability. Bars and error bars show mean and SE (n = 7). Different lowercase letters indicate significant difference among treatments (p< 0.05). Asterisks (*) indicate significance at p < 0.05.

In comparison to the CK treatment, the 3% SBC treatment showed a significant increase of 41.41% in community stability, and the 5% SBC and 5% FBC treatments showed increases of 22.13% and 20.14% respectively. However, it was notably reduced by 19.50% in the 3% FBC treatment (**Figure 5A**). Moreover, the findings from the unary linear regression analysis revealed a positive association between community stability and both the diversity of species and functional diversity (**Figures 5B, C**).

### 3.5 The mechanism of factors on plant communities

The results of spearman correlation analysis revealed that species abundance of communities was significantly negative affected by soil pH, salinity, SOM, AK, AP and TN. Communities height was significantly positive with soil TP, but was significantly negatively correlated with soil pH. Stem diameter of communities was significantly positively correlated with soil NH_4_^+^-N, but was negatively correlated with soil AK. Additionally, soil SOM, AK and TN were significantly positive with SRL and SRSA of community, but they were significant negative with RTD. Furthermore, community biomass and species diversity were negatively associated with soil pH. FDis and ICV were significantly positive with soil SOM. However, FDiv and RaoQ had no significant correlation with any of the soil physicochemical properties (**Figure 5D**).

The RDA was to investigate how soil properties influence the composition of plant communities (**Figure 5E**). The plot illustrated that the examined soil variables account for 39% of the total variation. The first axis explained 35% of the variation, while the second axis explained 4%. The vectors clearly distinguished the control treatments on the upper side of the graph from the biochar addition treatments, which are concentrated on the lower and right sides. Soil pH showed negative correlations with species composition in the CK group. However, soil pH exhibited positive correlations with species composition in the 1% JBC, 1% SBC and 1% FBC treatments, while soil SOM showed the opposite trend. Soil salinity was found to be positively associated with the species community in the 3% SBC treatment. Conversely, species composition in the 5% JBC treatment showed a negative relationship with soil salinity.

**Figure 6:**
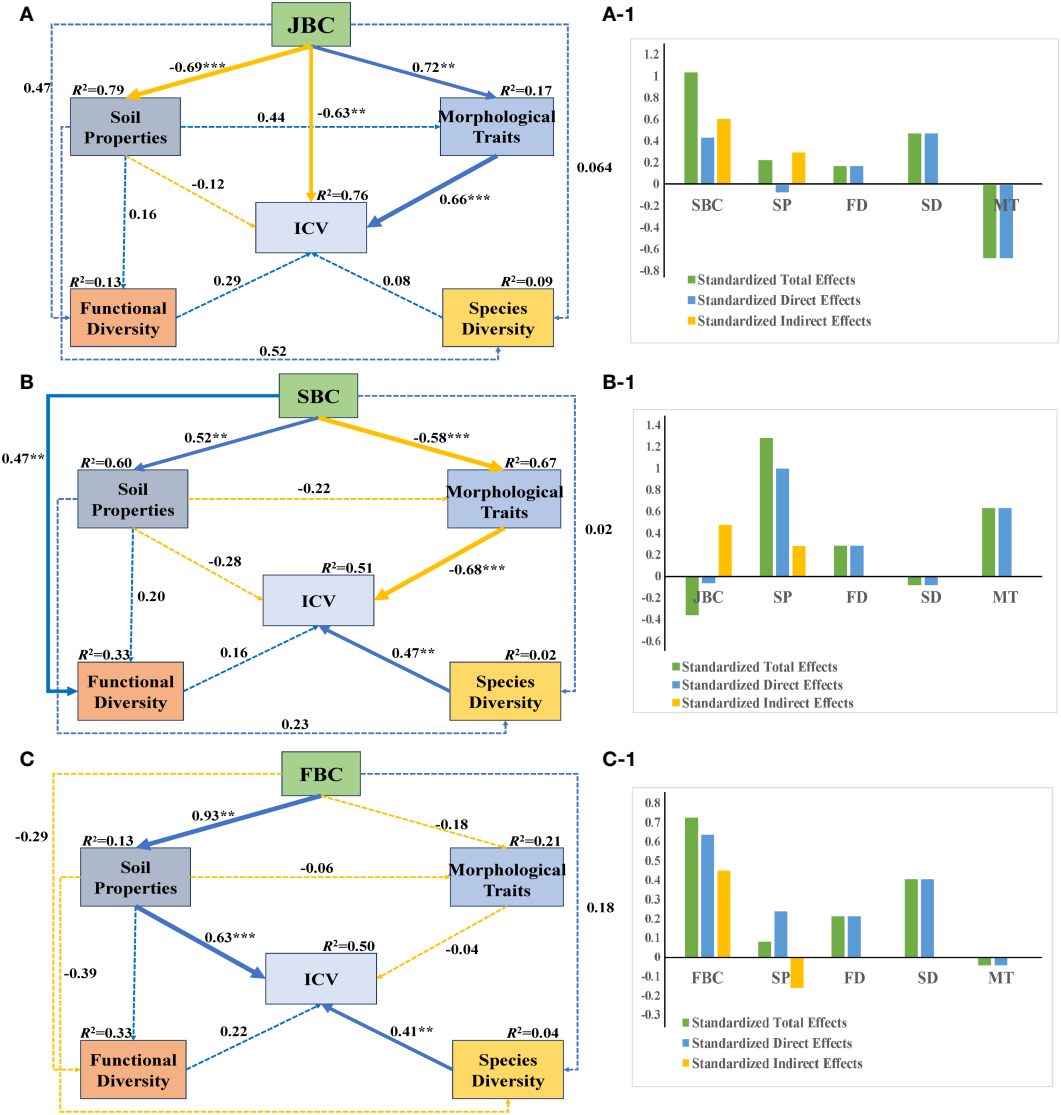
SEM analysis of the relationship among soil properties, morphological traits, species diversity, functional diversity and community stability under the three biochar addition conditions. Standardized path coefficients are shown next to the arrows, with arrow size indicating the strength of the coefficients. Positive paths are represented by blue lines, while negative paths are represented by orange lines. Solid lines indicate significant relationships (*p* < 0.05), while dashed lines indicate insignificant relationships (p > 0.05). The significance level is denoted by asterisks: ** *p* < 0.01, *** *p* < 0.001. The R2 values indicate the proportion of the variation explained by the relationships with other variables. Goodness-of-fit statistics for the A: Chi/DF = 1.105, GFI = 0.924, RMSEA = 0.069. Goodness-of-fit statistics for the B: Chi/DF = 1.076, GFI = 0.916, RMSEA = 0.085. Goodness-of-fit statistics for the C: Chi/DF = 1.168, GFI = 0.906, RMSEA = 0.079. **Figures A-1, B-1, C-1** show the standardized total effects, standardized direct effects and standardized indirect effects obtained from the SEM. SP, soil properties; FD, functional diversity; SD, species diversity; MT, morphological traits.

Under JBC biochar condition, JBC had significantly negative influences on soil properties and ICV (path coefficient = -0.69 and -0.63), while positively affecting morphological traits (path coefficient = 0.72), respectively. Additionally, morphological traits had a significant positive effect on ICV (path coefficient = 0.66) (**Figure 6A**). Soil properties shown the greatest standardized total and standardized direct effects on ICV, and JBC exhibited the largest standardized indirect effects on ICV (**Figures 6A-1**).

Under SBC biochar condition, SBC addition had a noticeable negative effect on morphological traits (path coefficient = -0.58), but it had a positive influence on soil properties and functional diversity (path coefficient = 0.52 and 0.47), respectively. Furthermore, morphological traits had a significantly negative impact on ICV (path coefficient = -0.68), while species diversity had a significantly positive effect on ICV (path coefficient = 0.47) (**Figure 6B**). SBC addition demonstrated the greatest standardized total and standardized indirect effects on ICV (**Figures 6B-1**).

Under FBC biochar condition, the SEM analysis showed (**Figure 6C**) that FBC addition had a significantly positive impact on soil properties (path coefficient = 0.93). Moreover, both soil properties and morphological traits shown positive effects on ICV (path coefficient = 0.63 and 0.41). FBC addition exhibited the biggest standardized total, standardized indirect and standardized indirect effects on ICV (**Figures 6C-1**).

## 4 Discussion

### 4.1 biochar change soil properties

In our study, we observed that biochar addition improved the soil pH (**Figures 1A, C, D**), which was consistent with previous researches (Biederman and Harpole, 2013; Akhtar et al., 2015). The alterations in soil pH after biochar addition are influenced by the initial pH of the biochar, which is usually alkaline (JBC pH: 9.84; SBC pH: 9.63; JBC pH: 9.80). The positive effects of biochar addition on pH in saline-alkali soil could be attributed to biochar contains more salt-based ions, such as Mg^2+^ and Ca^2+^. These ions can potentially decrease the levels of exchangeable aluminum ions and exchangeable hydrogen ions in the soil (Xu et al., 2014, Xu et al., 2017). Additionally, soil salinity was greatly reduced in treatments with 1% JBC, 5% JBC, 1% SBC and 5% FBC compared to the control treatment (**Figure 1B**). This reduction can be attributed to the porous structure and high specific surface area of biochar, which enhances the adsorption of Na^+^、Mg^2+^、Ca^2+^ in saline soil (Thomas et al., 2013; Akhtar et al., 2015; Hammer et al., 2015). The addition of biochar has the potential to enhance aggregate stability in soils, which can lead to reduced water evaporation and limited upward movement of saltwater. As a result, this can help in reducing salt accumulation in the topsoil (Yuan et al., 2023). However, it was observed that there was no significant change in soil salinity from CK for 3% SBC and 5% SBC treatments (**Figure 1B**), which could possibly be attributed to the fact that *S. alterniflora* is a halophyte (Cai et al., 2020; Wang et al., 2024). Hence, to prevent soil salinization during the application of SBC, it is necessary to desalt before carbonizing *S. alterniflora*.

As for soil nutrients, AK serves as a commonly used indicator for evaluating soil health. There is considerable evidence supporting the fact that biochar can increase AK levels in salt-affected soils (Biederman and Harpole, 2013; Akhtar et al., 2015; He et al., 2020). Biochar increased the content of AK in saline soils, primarily improving nutrient retention. This was due to its unique nutritional profile, as biochar is rich in potassium salts. Additionally, biochar could function as a fertilizer by releasing the soil nutrients that were initially present in the biomass (Gul and Whalen, 2016; Tang et al., 2020). However, the effects of adding biochar to alkaline soils can differ, leading to either a positive, negative, or no significant change (Xu et al., 2014; Bornø et al., 2018; Liang et al., 2021). The results of our study indicate that soil AP, NH_4_^+^-N, TP and TN did not show any increase with biochar additions, except for a significant increase in soil TP at 5% FBC (**Figures 1E, G, H**). The lack of increase in soil P nutrients probably due to the sorption or precipitation of P with biochar. In alkaline soils, biochar contains a substantial amount of free Ca^2+^, Al^3+^, Mg^2+^ and Fe^3+^ oxides, which could potentially serve as P sorption sites (Ngatia et al., 2017; Bornø et al., 2018). The correlation analysis also showed a significant negative relationship between soil P nutrients and soil pH (**Supplementary Figure S2**). This is in line with previous reports that have shown an increase in biochar P sorption capacity with higher pH levels in alkaline soils (Xu et al., 2014; Ngatia et al., 2017). In this study, the application of 5% FBC was found to be the most suitable option for saline-alkali soil, as it effectively reduced soil salinity while also increasing soil nutrient levels. This was likely due to the higher levels of sulfur and nitrogen elements in FBC (**Supplementary Table S1**), which provided essential nutrients for the soil microbial community and impacted the soil nutrient cycling. Taheri et al. (2023) also found the biochar with higher sulfur enhances the efficiency of biochar in amending saline and calcareous soil.

### 4.2 biochar change plant community morphological traits

The effects of biochar addition on plants are highly influenced by the diverse characteristics of biochar types and soil properties (Roberts et al., 2015; Gundale et al., 2016; He et al., 2020). Our study indicated that the addition of biochar not conducive to plant growth, which significantly decreased species abundance, stem diameter and height in biochar treatment by comparing the original soil sample (**Figures 2A–C**). According to the results of correlation analysis, soil pH was negatively associated with the species abundance and height of communities (**Figure 6**). This can be attributed that the seeds are not well-suited to high soil pH conditions, either failed to germinate or experienced post-germination mortality (Ma et al., 2012). Additionally, the toxic elements and soil microbial communities were also affected by increasing soil pH, further impacting the growth of plants (Weaver and Hamill, 1985). Our findings also revealed that the addition of biochar resulted in a decrease in plant community biomass as compared to the control treatment (**Figures 2D–F**), which opposes previous studies (Hammer et al., 2015; He et al., 2020). These unfavorable effects of biochar on plant community biomass were highly linked to soil pH and AP according to correlation analysis (**Figure 6**). Therefore, it indicates that soil pH and soil nutrients are important factors influencing plant biomass and quality in degraded coastal soils (Xu et al., 2020; Cui et al., 2021). Another possible explanation for the decline in community biomass could be the reduction in morphological traits and Shannon’s diversity (**Supplementary Figure S3**) (Token et al., 2022; Guo et al., 2023). Comparing different biochar treatments, it was observed that the community biomass in all SBC treatments depicted the lowest values (**Figures 2D–F**). This decline can primarily be attributed to the species abundance, stem diameter, height, RTD and Shannon’s diversity has declined on a large scale with SBC treatment. The negative effects of SBC on plants were found to be the most severe, potentially due to the simultaneous increase in soil pH and salinity after SBC addition. The combination of highly alkaline and saline environments worsens the harm caused by saline-alkali soil to plants, ultimately leading to a reduction in their morphology traits. The findings of this study suggest that there may be ecological risks when using biochar in salt-alkaline soils. Therefore, it is necessary to address these potential risks and explore various means to enhance plant health in salt- alkaline soils, such as utilize modification or combined application with other amendments to develop functional biochars (Cui et al., 2021; Zhang et al., 2022).

However, SRL and SRSA of plant communities were significantly improved at a middle and higher SBC biochar addition ratio (**Figures 2G, H**). The improvement in SRL and SRSA could be attributed to the fact that biochar can reduce the mechanical resistance to plant root growth by altering the soil composition, ultimately promoting the growth of plant roots (Li et al., 2021). Additionally, correlation analysis also revealed that SRL and SRSA of communities were negatively associated with the species abundance (**Supplementary Figure S3**), one potential explanation for it may be that the lesser species abundance could alleviate space stress and optimize the space for plants root growth, further provide a more favorable environment for root elongation.

### 4.3 biochar change community composition and community diversity

**Figure 7:**
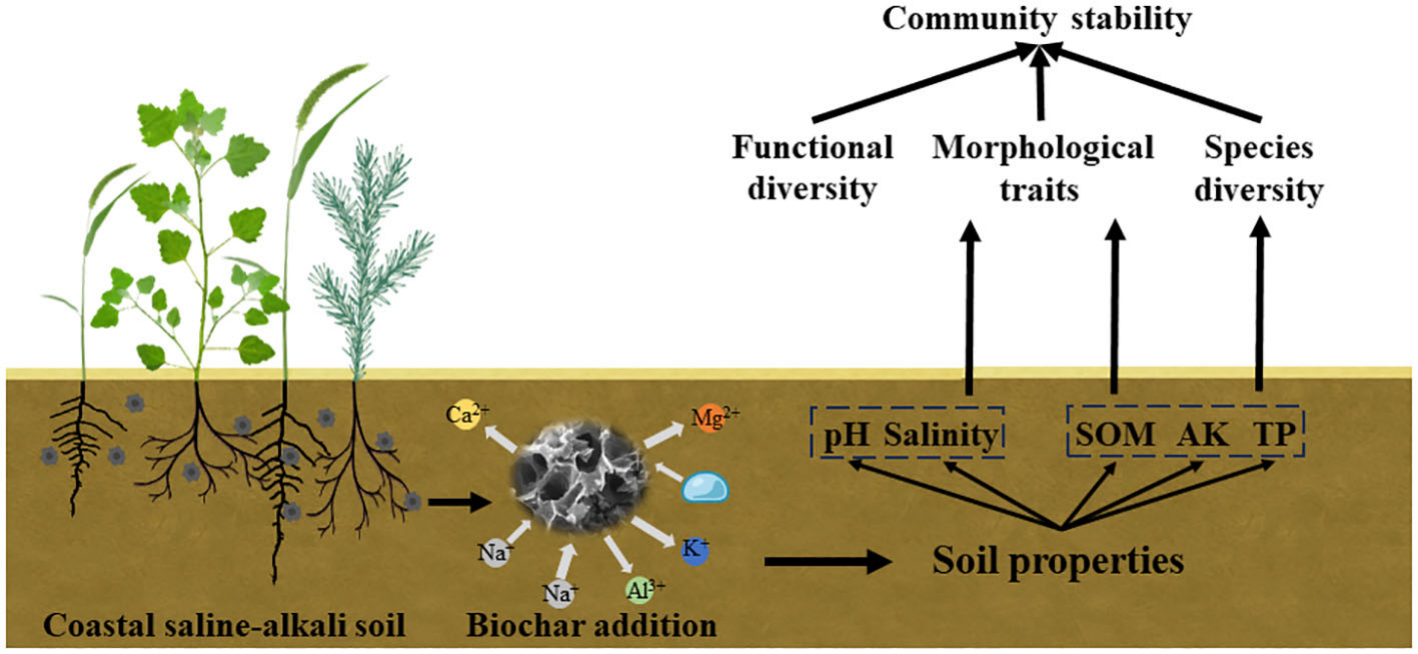
The mechanism of biochar addition on community stability in saline-alkali soil.

The results of the NMDS and ANOSIM analysis indicate that JBC, SBC and FBC biochar addition can significantly modify the composition of plant species when compared to the CK treatment (**Figure 3**; **Supplementary Table S3**). Due to the fact that species inside the community will co-occur and compete with each other, the alterations of soil properties brought by biochar application will inevitably change the species composition (Roberts et al., 2015; Gundale et al., 2016). The RDA analysis revealed distinct assemblages of species depending on the types and ratios of biochar used (**Figure 7**), where soil pH and SOM exhibited correlations with plant species composition in the 1% JBC, 1% SBC and 1% FBC treatments. Soil salinity was found to be associated with the species community in the 3% SBC and 5% JBC treatment. This could be attributed to the success of seed germination is heavily influenced by the variations in soil nutrient levels, which are determined by the different types of biochar used. Seeds that adapted to these specific environmental conditions have a higher probability of thriving and successfully germinating (Farooq et al., 2020; Cui et al., 2021);. According to the species diversity of plant communities (**Figures 4A–C**), the Shannon-Weiner diversity index, Simpson diversity index and Pielou’s evenness index showed a significant decrease in most biochar treatments. Correlation analysis demonstrated a negative association between soil pH and species diversity (**Figure 6**). These findings differ from several other studies that reported significant positive correlations between species diversity and pH in acidic soil (Chytrý et al., 2003; Schuster and Diekmann, 2003). The reduction in species diversity can be attributed to the inhibitory effects of increasing pH on seed viability and germination, especially in alkaline soils (Ma et al., 2012). Specifically, alkaline-tolerant plants such as *Suaeda salsa*, *Bassia scoparia*, *Setaria viridis*, *Chenopodium album*, and *Polygonum aviculare* tend to thrive in environments with higher pH levels. This can ultimately lead to a decrease in species diversity.

According to the functional diversity of plant communities, the FDis index, FDiv index and RaoQ index were also changed by biochar addition (**Figures 4D–F**). Correlation analysis proved that soil SOM played a crucial role in influencing the FDis index (**Figure 6**), which was consistent with previous observations that improved soil nutrients could improve plant morphological traits (Liang et al., 2021; You et al., 2021). However, we also discovered that FDis index and RaoQ index were decreased significantly at 3% JBC, 1% SBC and 3% FBC treatments, which possible connection with the decline in community species diversity (**Supplementary Figure S3**) (Mayfield et al., 2010).

### 4.4 biochar change plant community stability

Ecosystem stability plays a vital role in biodiversity conservation and sustainable development. Recent studies have shown that the stability of communities is influenced by environmental stress, interspecific competition and interference activities (Lv et al., 2007). The analysis revealed differences in community stability among different biochars. Specifically, community stability was found to be significantly higher in 3% SBC, 5% SBC and 5% FBC compared to the other biochars (**Figure 5A**). This enhancement was linked to alterations in species diversity, functional diversity and variations in soil properties (**Figures 5B–D**). We found a significant positive correlation between species diversity and community stability (**Figure 5B**), but the results showed that the correlation coefficient of Pielou’s evenness with community stability was significantly greater than that of dominant species (**Supplementary Figure S3**). The addition of 3% SBC, 5% SBC and 5% FBC decreased the relative abundance of dominant species compared with other biochar. According to the “Complementary Effect”, an increase in biodiversity promotes complementary resource utilization through niche complementation (Wang et al., 2019; Jiang et al., 2022; Token et al., 2022). Moreover, we also found the positive correlation between the FDis index and community stability. Functional dispersion serves as an indicator of niche complementarity, with higher values reflecting stronger competition and niche complementarity among species (Jiang et al., 2022). The increase in functional dispersion contributes to maintaining community stability. Furthermore, the stability of plant communities is inevitably influenced by environmental factors as plants are closely linked to their surroundings. Specifically, SOM, AK, and TP exhibited a significant positive correlation with community stability (**Figure 6**). Soil factors can influence community stability through direct and indirect effects. The direct impact could involve increased soil nutrient absorption by plants after biochar application, enabling species to occupy more ecological niches. The indirect effect may involve the provision of more favorable soil conditions for non-dominant species, hereby improving the evenness.

SEM reveals that the leading drivers of community stability differed by different biochar types. Soil properties and morphological traits were identified as the primary drivers of community stability in JBC treatments (**Figure 6A**). Conversely, for the SBC treatments, morphological traits and species diversity were found to be significantly related to community stability, and community stability in FBC treatments were found to be driven by soil properties and species diversity (**Figures 6B, C**). These variations in the drivers of community stability can likely be attributed to changes in the soil environment (Lv et al., 2007; He et al., 2019). What’s more, we also observed that community biomass was negatively associated with community stability (**Supplementary Figure S3**). This suggests that a low biomass does not necessarily indicate a low level of community stability. In fact, some communities with simpler structures and single populations tend to exhibit higher levels of stability (Li et al., 2008). Furthermore, it is important to emphasize the scale at which plant community stability is assessed. In future studies, our intention is to broaden the scope of our research by investigating the impacts of biochar addition in field conditions on the stability of the community.

## 5 Conclusion

Research findings have indicated that the addition of biochar into soil has the potential to enhance soil pH and AK, as well as reduce soil salinity (expect for 3% SBC and 5%SBC). However, it has less impact on N and P nutrients. Among the different biochar types, the application of 5% FBC was found to be the most effective in increasing nutrients and reducing salinity. The addition of biochar has been found to significantly reduce the abundance of plant species, height, stem diameter, biomass and RTD of plant communities. In particular, the negative effects of SBC on plants were found to be the most severe. The findings suggested that the plant community performance was primarily affected by soil pH, salinity and nutrients levels. However, root specific length (SRL) and root specific surface area (SRSA) of the plant community were increased in 3% SBC and 5% SBC treatments. Moreover, the species diversity and functional diversity of plant communities can be altered by biochar, ultimately impacting community stability. The negative findings of this study suggest that there may be ecological risks when using biochar in salt-alkaline soils. Furthermore, to prevent soil salinization during the application of SBC, it is recommended to desalt before carbonizing *S. alterniflora*.

## Data availability statement

The original contributions presented in the study are included in the article/**Supplementary Material**. Further inquiries can be directed to the corresponding author.

## Author contributions

ZW: Writing – original draft, Writing – review & editing. MH: Writing – review & editing. XL: Writing – review & editing. ZM: Writing – review & editing. JL: Writing – review & editing. XM: Writing – review & editing.
