# Supplementary material for: Biochar addition can negatively affect plant community performance when altering soil properties in saline-alkali wetlands
Source: Front Plant Sci. 2024 May 16;15:1347658. doi: 10.3389/fpls.2024.1347658 (PMC11137290; doi:10.3389/fpls.2024.1347658)
Supplement: Supplementary file 1 [file DataSheet_1.docx]

Supplementary Information for

**Biochar addition can negatively affect plant community performance when altering soil properties in saline-alkali wetlands**

Ziyi Wang^a^, Mengxuan He^a^, Xueqiang Lu^b^, Zirui Meng^a^, Jie Liu^c^, Xunqiang Mo^a*^

^a^ School of Geographic and Environmental Science, Tianjin Normal University, Tianjin 300387, China

^b^ College of Environment Science and Engineering, Nankai University, Tianjin 300350, China

^c^ State Key Laboratory of Herbage Improvement and Grassland Agro-ecosystems, Center for Grassland Microbiome, College of Pastoral Agriculture Science and Technology, Lanzhou University, Lanzhou 730020, China

* Corresponding author.

Xunqiang Mo, School of Geographic and Environmental Science, Tianjin Normal University, Tianjin 300387, China

E-mail address: [421973@163.com](mailto:421973@163.com)

**Table S1.** Physicochemical properties of JBC, SBC and FBC pyrolyzed at 550℃ (Meng et al., 2023).

|  |  | **JBC** | **SBC** | **FBC** |
| --- | --- | --- | --- | --- |
| **pH** |  | 9.84 | 9.63 | 9.80 |
| **Elemental composition (%)** | C | 62.92 | 35.81 | 52.09 |
|  | H | 2.75 | 4.55 | 2.05 |
|  | O | 12.23 | 22.08 | 11.74 |
|  | N | 0.18 | 0.67 | 0.49 |
|  | S | 0.01 | 0.38 | 0.71 |

**Table S2.** Root morphological traits of plant communities. Values represent means (n = 7) and standard error. Different lowercase letters indicate significant difference among treatments (p < 0.05).

| **Group** | **Root_Length** | **Root_ProjArea** | **Root_SurfArea** | **Root_AvgDiam** | **Root_RootVolume** | **Root_Tips** |
| --- | --- | --- | --- | --- | --- | --- |
|  | **cm** | **cm^2^** | **cm^2^** | **mm** | **cm^3^** |  |
| **CK** | 1501.7±163.14a | 87.35±11.67a | 274.4±36.66a | 0.58±0.04e | 4.07±0.7a | 6612±444.74a |
| **1%JBC** | 248.09±24.65c | 22.55±3.03de | 70.85±9.53de | 0.9±0.06ab | 1.65±0.3de | 1585.57±111.42de |
| **3%JBC** | 980.88±134.15b | 67.72±11.5ab | 212.76±36.12ab | 0.67±0.04de | 3.75±0.77ab | 3869.43±433.82b |
| **5%JBC** | 826.89±160.97b | 51.9±8.21bc | 163.04±25.78bc | 0.68±0.07de | 2.68±0.4bcd | 3220.29±473.21bc |
| **1%SBC** | 240.57±110.02c | 18.24±7.97de | 57.31±25.04de | 0.88±0.08abc | 1.13±0.46e | 959.71±266.94ef |
| **3%SBC** | 97.48±22.56c | 7.06±1.8e | 22.19±5.65e | 0.74±0.07bcde | 0.42±0.12e | 338.29±64.45f |
| **5%SBC** | 382.1±98.44c | 22.94±4.76de | 72.07±14.96de | 0.71±0.08cde | 1.13±0.18e | 1626.71±302.71de |
| **1%FBC** | 1017.93±265.43b | 61.27±13.64b | 192.5±42.87b | 0.68±0.07de | 3.01±0.57abc | 3923±787.1b |
| **3%FBC** | 328.17±84.66c | 26.09±7.18de | 81.96±22.57de | 0.8±0.05abcd | 1.68±0.52cde | 1718.14±362.71de |
| **5%FBC** | 377.58±37.39c | 35.89±3.1cd | 112.76±9.73cd | 0.96±0.02a | 2.69±0.22bcd | 2157±254.52cd |

**Table S3.** Analysis of similarities on plant communities.

| **P**  **R** | **CK** | **1%JBC** | **3%JBC** | **5%JBC** | **1%SBC** | **3%SBC** | **5%SBC** | **1%FBC** | **3%FBC** | **5%FBC** |
| --- | --- | --- | --- | --- | --- | --- | --- | --- | --- | --- |
| **CK** |  | 0.0252 | 0.0209 | 0.0218 | 0.0006 | 0.0008 | 0.0005 | 0.0157 | 0.0004 | 0.0008 |
| **1%JBC** | 0.2352 |  | 0.3524 | 0.4253 | 0.0014 | 0.001 | 0.0008 | 0.9478 | 0.0072 | 0.0004 |
| **3%JBC** | 0.31 | 0.004373 |  | 0.0584 | 0.0005 | 0.0008 | 0.0005 | 0.6443 | 0.0016 | 0.0007 |
| **5%JBC** | 0.2337 | -0.00194 | 0.1594 |  | 0.003 | 0.0008 | 0.0004 | 0.6384 | 0.0038 | 0.0007 |
| **1%SBC** | 0.7235 | 0.4781 | 0.5889 | 0.5121 |  | 0.0005 | 0.0025 | 0.001 | 0.5268 | 0.0005 |
| **3%SBC** | 0.9903 | 0.9276 | 0.9388 | 0.9611 | 0.9572 |  | 0.0089 | 0.0001 | 0.0008 | 0.0006 |
| **5%SBC** | 0.8479 | 0.7619 | 0.8377 | 0.8047 | 0.4193 | 0.4169 |  | 0.0007 | 0.0015 | 0.0016 |
| **1%FBC** | 0.2425 | -0.1001 | -0.06074 | -0.03304 | 0.5768 | 0.9475 | 0.8105 |  | 0.0021 | 0.0008 |
| **3%FBC** | 0.6016 | 0.3124 | 0.5073 | 0.3513 | -0.03013 | 0.9602 | 0.4951 | 0.4184 |  | 0.001 |
| **5%FBC** | 0.6414 | 0.5811 | 0.5598 | 0.6497 | 0.793 | 0.861 | 0.7716 | 0.6453 | 0.7707 |  |

**Table S4.** The goodness-of-fit statistics of the model in RDA analysis.

| **Name** | **Explains %** | **Contribution %** | **pseudo-F** | ***p*** |
| --- | --- | --- | --- | --- |
| **SOM** | 29 | 70.1 | 27.8 | **< 0.01** |
| **Salinity** | 4.4 | 10.7 | 4.5 | **< 0.05** |
| **pH** | 3.6 | 8.6 | 3.7 | **< 0.05** |
| **AH** | 1.2 | 2.9 | 1.3 | > 0.05 |
| **TP** | 1 | 2.4 | 1.1 | > 0.05 |
| **AP** | 0.8 | 1.9 | 0.8 | > 0.05 |
| **AK** | 0.9 | 2.3 | 1 | > 0.05 |
| **TN** | 0.4 | 1.1 | 0.5 | > 0.05 |


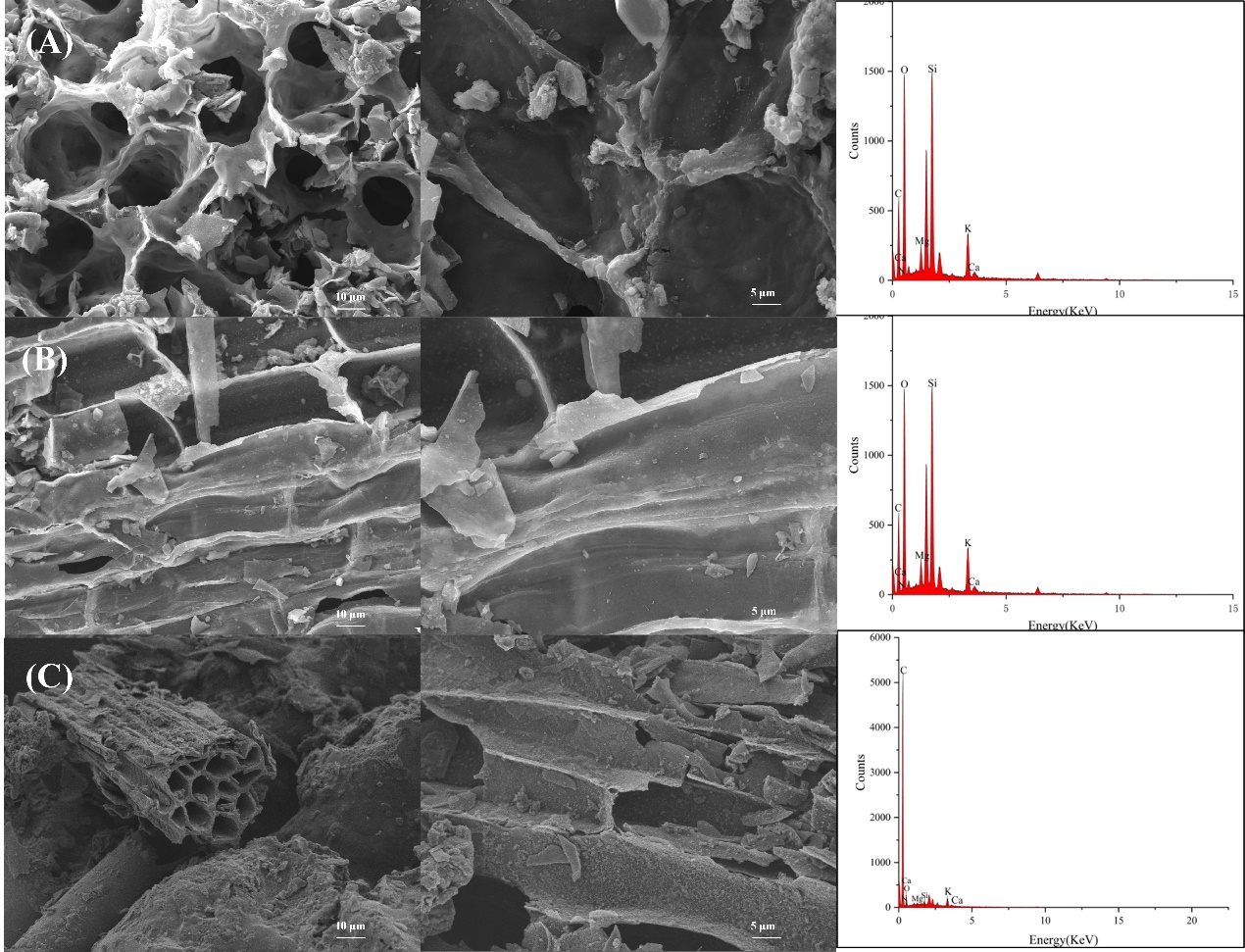


**Fig. S1.** Scanning electron microscope (SEM) images and corresponding energy dispersive spectroscopy (EDS) spectra of (A) *Juglans regia* biochar, (B) *Spartina alterniflora* biochar and (C) *Flaveria bidentis* biochar pyrolyzed at 550 ℃ (Meng et al., 2023).

**

**

**Fig. S2.** Spearman correlation heatmaps between physical and chemical properties of soil. TN: total nitrogen; TP: total phosphorus; AN: ammonia nitrogen; AP: available phosphorus; AK: available potassium; SOM: soil organic matter; Salinity: soil salinity. Asterisks (*) indicate significance at *p* ＜ 0.05.


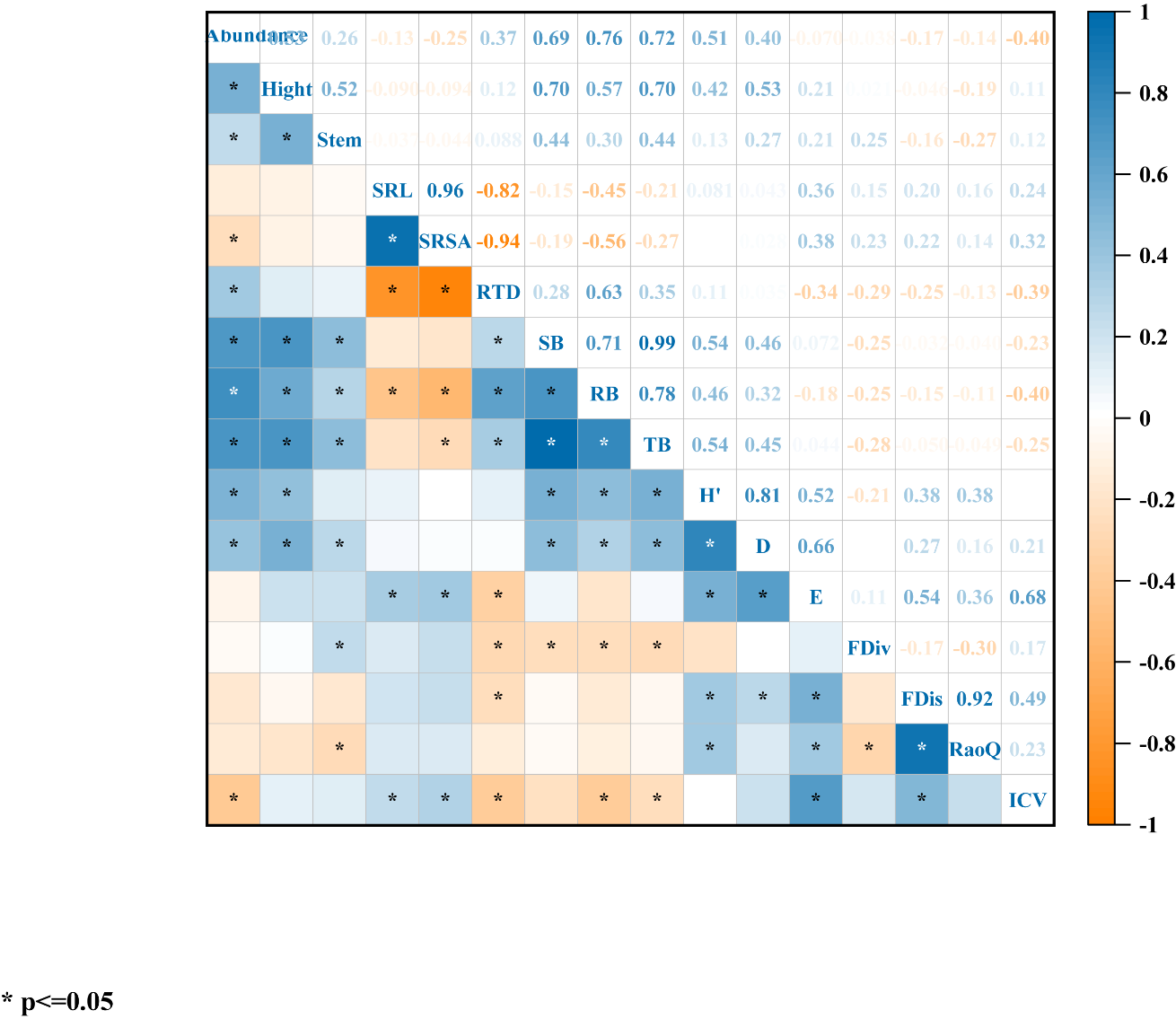


**Fig. S3.** Spearman correlation heatmaps between morphological traits, species diversity, functional diversity and community stability. SRL: specific root length; SRSA: specific root surface area; RTD: root tissue density; SB: shoot biomass; RB: root biomass; TB: total biomass; H': Shannon-Wiener diversity index; D: Simpson diversity index; E: Pielou’s evenness index; FDiv: Function divergence index; FDis: Function dispersion index; RaoQ: Rao’s quadratic entropy index; ICV: community stability. Asterisks (*) indicate significance at *p* ＜ 0.05.

**

**

**Fig. S4.** Effects of different biochar addition on plant community structure.

# Reference

Meng, Z., Mo, X., Meng, W., Hu, B., Li, H., Liu, J., Lu, X., Sparks, J.P., Wang, Y., Wang, Z., He,

M., 2023. Biochar may alter plant communities when remediating the cadmium-contaminated soil in the saline-alkaline wetland. *Sci. Total Environ.* **899**, 165677.
